# Orthodontic Bracket Removal Using LASER-Technology—A Short Systematic Literature Review of the Past 30 Years

**DOI:** 10.3390/ma15020548

**Published:** 2022-01-12

**Authors:** Anca Mesaroș, Michaela Mesaroș, Smaranda Buduru

**Affiliations:** 1 Department of Prosthodontics and Dental Materials, Iuliu Hațieganu University of Medicine and Pharmacy, 400012 Cluj-Napoca, Romania; dana.buduru@umfcluj.ro; 2 Department of Pediatric Dentistry, Iuliu Hațieganu University of Medicine and Pharmacy, 400012 Cluj-Napoca, Romania; michaela.mesaros@umfcluj.ro

**Keywords:** laser, orthodontics, bracket removal, debonding

## Abstract

Background: Since fixed orthodontic treatment is widely spread and one of its inconveniences is bracket removal, as this affects enamel integrity as well as being a cause of discomfort to the patient, studies have searched for the most adequate bracket removal technique, many of them focusing on using laser-technology. Methods: Our review focused on articles published investigating methods of orthodontic bracket removal using laser technology in the last 30 years. Results: 19 relevant studies were taken into consideration after a thorough selection. Different types of laser devices, with specific settings and various testing conditions were tested and the investigators presented their pertinent conclusions. Conclusions: Most studies were performed using ceramic brackets and the best results in terms of prevention of enamel loss, temperature stability for the tooth as well as reduced chair time were obtained with Er:YAG lasers.

## 1. Introduction

In present times, orthodontic treatment is highly sought by patients of all ages. The most common orthodontic treatment consists in bonding brackets or other attachments to dental structures to move teeth. The process of bonding attachments to enamel involves several steps, starting with acid etching which has the purpose of creating microporosities that favors the micro-retention of resin infiltrating into the enamel [1]. When the active orthodontic treatment is finished, the brackets used are de-bonded and all adhesive remnants should also be removed since residual adhesive favors the accumulation of dental plaque and might also lead to discoloration [2]. As demonstrated by numerous studies, no mechanical technique available to the practitioners allows debonding without any damage whatsoever to the enamel surface [3]. Also, the mechanical debonding procedures can cause pain and discomfort for the patients [4]. With the development of technology, alternative techniques for orthodontic bracket removal have been tested.

The term laser is an abbreviation of “light amplification by stimulated emission of radiation” [5]. In the 1980s oral surgeons started using carbon dioxide (CO_2_) lasers on soft tissue and thus the first use of lasers in dentistry occurred. Since then, many innovations and developments in both the medical and laser-technology fields have followed until the present. In 1997, the United States Food and Drug Administration (FDA) permitted the use of laser-devices with erbium for procedures on hard tissue and in 1998 the use of the first diode laser for procedures on soft tissue was allowed [6].

Laser techniques are now also available for use during orthodontic treatment and studies have proved their use to be advantageous [7,8,9,10]. Soft tissue lasers are often used for procedures such as gingivectomy and frenectomy of surgical exposure of impacted teeth because they inhibit bleeding and swelling, offer an improved cut precision, reduce the pain for the patient and prevent to some degree wound contraction [5,6,11,12,13]. Other applications of lasers in orthodontics refer to enamel etching and bonding and bracket debonding [14,15,16]. Also, low-level laser therapy has great potential for pain control and accelerating orthodontic tooth movement [17,18,19].

The use of laser technology allows less force application throughout the removal process of brackets because of the softening within the adhesive resin material that was originally used to bond the brackets [20].

Laser energy helps remove the adhesive resin from the tooth surface in three ways: thermal softening, thermal ablation and due to the effects of photoablation. Despite the effectiveness of this method, it also presents some inconveniences such as the occasional diffusion of the heat to the tooth structure, which can lead to pulpal damage [21,22,23]. Studies have shown that a 5.5 °C rise of the temperature inside the pulpal cavity can induce pulpal necrosis, nevertheless other studies have proved that the use of diode lasers with either 1 W or 3 W power for 3 s can effectively debond the ceramic brackets without inducing detrimental effect on the enamel nor inside the pulp cavity [24]. 

Throughout the years, different studies were conducted to investigate the applicability of various laser types and settings for orthodontic attachment removal [20]. Many of them were performed using carbon dioxide (CO_2_), diode, erbium-doped yttrium aluminum garnet (Er:YAG) and neodymium-doped yttrium aluminium garnet (Nd:YAG) lasers, etc. 

Debonding procedures of orthodontic attachments using laser-technology are especially, but not exclusively, used for ceramic brackets as they have a stronger adhesion with the enamel, and in particular when using Er:YAG lasers in scanning mode which decreases the shear bond strength [20].

The use of Er:YAG lasers together with water cooling seems to represent a safer option for reducing the chance of intrapulpal temperature increases while effectively debonding ceramic brackets by reducing resin shear bond strength [22,24].

The aims of this review are:

- to provide a comprehensive literature review of the available methods for orthodontic bracket removal using laser-technology
- to search for the best approach of using laser-technology for orthodontic bracket debonding with minimal risks for patients by answering the following questions:
  ◦ What would be the best parameters such as energy or frequency of the laser for a safe debonding procedure?
  ◦ What effects does the procedure have on hard dental tissues such as enamel or soft tissues such as the pulp?
  ◦ Is the shear bond strength between bracket and substrate affected, and if yes, in what way?

## 2. Materials and Methods

The PubMed, Web of Science, Springer and Scopus databases were searched from inception to June 2021 for studies on the use of laser technology for orthodontic bracket debonding. The search was carried out using the keywords “laser debracketing”, ”laser and orthodontic bracket removal” and “laser and orthodontic debonding”. The following search strategy was employed: ((laser debracketing) OR (laser and orthodontic bracket removal)) OR (laser and orthodontic debonding) Filters: Abstract, Full text, Clinical Trial, Meta-Analysis, Randomized Controlled Trial, Review, Systematic Review, from 1 January 1990–1 September 2021. 

The present review was constructed in accordance with the guidelines of the Preferred Reporting Items for Systematic Reviews and Meta-Analysis (PRISMA) Statement [25]. 

The articles selected for this review were subject to the following inclusion and exclusion criteria. Inclusion criteria: English language of publication, available in full text format and investigating laser-aided orthodontic bracket debonding. Reviews, case reports, comments, and letters to the editor were excluded. Lack of access to the full article or use of a different language than English disqualified studies for being taken into consideration.

After reading the abstracts and full texts of articles, the papers consistent with the review subject were selected. 

Articles selected for the review were using the laser-technology during the actual bracket debonding phase and all articles referring to composite removal after debracketing, or studies that investigated shear bond strength after exposure to laser were not considered eligible. Three independent reviewers contributed to the selection, inclusion and data-extraction process. During the assessments for full-text eligibility three conflicts arose and agreement was reached when two out of three votes were in agreement.

PICO study characteristics are presented as follows: Studies performed on human or animal natural teeth, the employed procedure is the use of laser-technology for removing orthodontic brackets from dental enamel, comparing different techniques (lasers, laser-settings), the removal of metallic or ceramic brackets and having as an end result the breakage of bonding between bracket and enamel. 

Studies that passed the eligibility criteria were classified by year of publishing, type of study conducted, type of laser-technology used and their most relevant results. Articles described multiple variables including temperature inside the pulp chamber, shear and tensile bond strengths, alterations of the enamel, debonding timing, and even potential bracket damage by breakage. 

As the debonding process is also affected by bracket type, laser type, lasing mode, lasing time and laser power, these parameters were also included in our study when possible. The 19 identified studies cover a time period from 1992 until 2020.

## 3. Results

**Figure 1:**
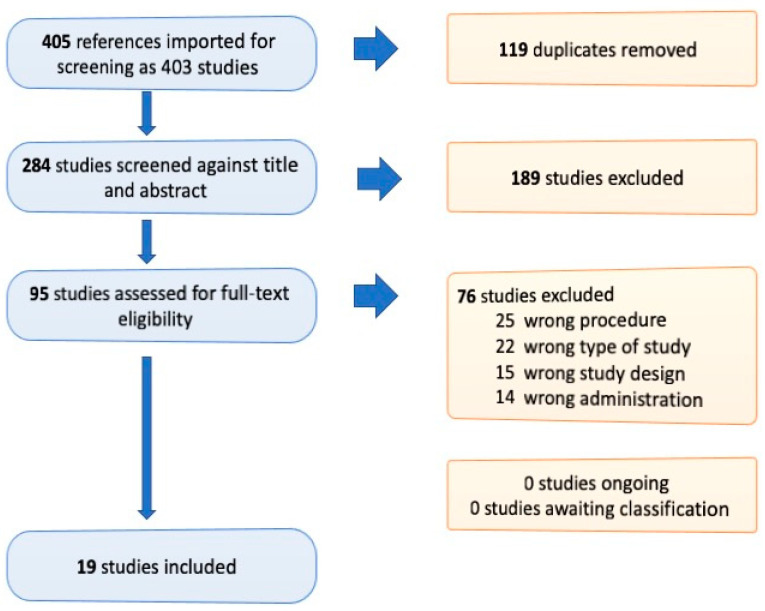
PRISMA Flow diagrame showing the selection of articles.

Our search revealed a total of 419 articles (69 in the PubMed database, 87 in Web of Science-All Databases” 220 in the Springer Link database and 43 in the Scopus Database, respectively). All references were imported into Zotero Research Assistant, 14 presented problems during the importation process and thus the study is based on 405 studies that were imported in Covidence.org—Systematic Review Software. The PRISMA Flow Diagrame presented in Figure 1 presents the selection process of articles after being imported into the software. 

Selected articles were separated by analysing the type of laser used in the described research. Thus, Table 1, Table 2, Table 3 and Table 4 present the most pertinent results of the study. Table 1 presents studies using CO_2_ laser-technology, Table 2 presents studies using Er:YAG laser-technology, Table 3 presents studies using Nd:YAG, Diode and fiber-laser technologies while Table 4 summarizes results from studies comparing different laser-technologies. In each of the tables, articles are presented in chronological order.

The studies using CO_2_ laser technology for orthodontic bracket removal show that this type of technology reduces significantly the bond strength while also avoiding a dangerous increase of temperature in the pulp chamber and thus protecting tooth vitality and preventing enamel damage.

The use of Er:YAG laser-technology shows similar results as the use of CO_2_ laser technology by also producing a decrease of bond strength, reduces risk of enamel damage, however increases of temperature are dependant on the lasing mode and device settings should be managed carefully in order to avoid pulp damage.

Nd:YAG, diode and fiber lasers also favour orthodontic bracket removal with reduced risk of damage to the enamel by diminishing bond strength.

Comparative studies between different laser technologies were done only using ceramic brackets, however the studies revealed that regardless of the technology used the results of debracketing were similar and superior to situations where no laser technology was employed. An interesting aspect of our study is that all articles included for this review were experimental in-vitro studies and none of them were clinical studies.

## 4. Discussion

The key factor in orthodontic bracket removal is diminishing the strength of the adhesive connecting the attachment to the tooth [41,42]. Such alteration can be achieved by laser radiation which penetrates the bracket towards the adhesive resin and thus influencing its bond’s strength to the enamel [38]. However, the power of penetration of the laser radiation is thus influenced by the material of which the bracket is made of [22]. Only three [22,30,33] of the studies included in our review also used metallic brackets, and only in two, the investigators have applied the laser radiation in combination with them [22,30].

Investigators concurred that changes within the adhesive resin due to the use of laser technology occur when the wavelength is transmitted through the bracket. Therefore, the CO_2_ laser—which has a wavelength that is more easily absorbed by ceramic brackets—has been chosen for investigation of debonding in some studies [26].

Other studies have shown that lasers of different types, such as Nd:YAG, Er:YAG, Diode and Tm:YAP (Tm^3+^-doped YAlO_3_) have been tested alone or in comparison with the purpose of debonding ceramic brackets from dental surfaces, and each of them have been proven efficient with its own advantages and limits [32,33,34,43]. 

Investigators have suggested that the direct application of the laser to the orthodontic adhesive resin would favor the effects of thermal ablation and photoablation, however, this procedure proves to be difficult in clinical settings as increased amounts of bonding agent are not desired. Nd:YAG laser was used for experiments in this direction, as it has a lower absorption in the bracket if compared to carbon dioxide lasers [28,33].

The advantages of the diode laser, such as its relatively small size, weight, adjustable power and acceptable cost make it a good choice for studies, as well as a smart choice for dental practices [44,45,46].

Laser technology using Er:YAG lasers presents some advantages when used for debracketing due to their versatility. A proper adjustment of their settings such as water/air concentration, power, used energy, frequency, time, and irradiation method is essential in protecting the integrity of the enamel surface and preventing the increase of temperature in the intrapulpal chamber beyond the acceptable thresholds [30,31,40].

The low tensile strength of enamel in combination with high debonding strengths of the orthodontic adhesive and associated with the low toughness against fracture of ceramic brackets all lead to enamel fractures and bracket failures.

Tocchio et al. [38] showed that when employing an Nd:YAG laser, energy traverses the bracket and it is either absorbed at the base or it is reflected due to the mechanical structure. The part of the energy that is absorbed will be transformed to localized thermal energy, resulting in one of the following situations:

- thermal softening of the adhesive
- thermal ablation(causing brackets to blow off)
- photo-ablation (also causes blowoff of the brackets) [29,38].

The three processes: thermal softening, thermal ablation and photoablation are mainly responsible for adhesion alteration during orthodontic bracket removal using laser-technology.

During the thermal softening process the orthodontic adhesive is heated and as a result its consistency softens until the bracket easily slides off the surface of the tooth.

Thermal ablation means that the temperature of the adhesive resin rapidly increases, and the substrate is blown off the surface of the enamel prior to the occurrence of thermal softening.

During the photoablation process, the atoms of the adhesive resin have energy levels that are rapidly increasing more than their dissociation energy levels determining fractures within the material because of the decomposition [24].

Thermal softening process happens at low power densities, while thermal ablation and photoablation occur at high power densities.

The pressure that appears as a result of the rapid thermal expansion and evaporation of the adhesive resin acts as the disconnecting force. It turns out that when the brackets are blown off and it takes more than 0.5 s for debonding to occur, it means that a single pulse was not enough and several pulses were required for thermal softening to occur and cause debracketing.

By the same logic, if debracketing happened by blowing off and it happened in less than 0.5 s, it means that thermal ablation or photoablation mechanisms are responsible for the debonding.

Hayakawa [32] presented the idea that the gas pressure which is generated by thermal ablation or photoablation acts as a disconnecting force that propagates evenly in the binding interface, allowing the brackets to detach.

Even if Tocchio [38] proved in 1993 that the use of an KrF-excimer laser source of 248 nm creates a photoablative debracketing process using only one pulse, other studies [29] show that Er:YAG lasers of 2.94 μm is a better choice for clinical use. While the use of an 248-nm excimer laser for medical purposes is prohibited as its emission in the UV band is the same with DNA absorption, the Er:YAG laser is well-appreciated for its versatility, an increased absorption in water of more than 10,000/cm allowing the debracketing to take place by a similar blowoff process without the risk of removing a great amount of adhesive resin from below the bracket and ensuring that thus preventing damage to the enamel [31].

As some studies showed that the use of laser technology in orthodontics can improve pain management during orthodontic tooth movement [45,47,48,49,50], or during surgical interventions on soft tissues for orthodontic purposes [12,51], our review also emphasizes the fact that by reducing the force needed for bracket debonding and by careful management of the increase in temperature within the pulp chamber, the orthodontic bracket removal by laser-technology also diminishes the pain and the discomfort of the patient during the debonding procedure.

Due to the fact that the spectral transmissibility within the polycrystalline brackets that are available on the market is low compared to the transmissibility within monocrystalline (sapphire) brackets, the densities of energy, as well as the pulse powers that are required from the lasers to obtain ablative debonding should be greater [42,52,53]. As the transmission within polycrystalline brackets increases with longer wavelengths, lasing using an emitting radiation between 4 to 7 I.tm could lead to ablatively debracketing without producing excessive heat. Using of adhesives designed to absorb light at specific wavelengths, can facilitate resin alteration at lower laser power levels and decrease the amount of thermal heating of the bracket [38].

Although in the last 30 years studies have been made to use lasers for orthodontic bracket removal, a relatively recent study based on a survey upon the preference for debonding and awareness of different and alternative debonding techniques showed that 83% of 119 orthodontists who responded to the questionnaire were not aware of debonding techniques of ceramic brackets using lasers while most orthodontists who use ceramic brackets in their practice used only mechanical debonding [54,55].

The present review includes studies from 1992 until present times. During this period of 30 years many developments have taken place in the field of laser applications, technologies have evolved, as well as research guidelines and protocols and so a thorough comparison of the presented studies with a qualitative assessment of them was considered to be subjective and biased.

The strengths of this review reside in the fact that while covering a long period of time, many types of lasers, with different settings were used. However, further research in clinical setting is to be desired, as eligible studies for this review used only in-vitro testing.

## 5. Conclusions

The use of various types of lasers during orthodontic bracket removal procedures has been proven to be beneficial. There is no clear indication that only one type of laser would be significantly a better or safer choice for the procedure of orthodontic bracket removal, nor is there a “recipe for success” in laser settings. This review consists of 19 in vitro experimental studies that prepare practitioners for the use of lasers in clinical everyday practice, however in-vivo studies, maybe even split-mouth studies should also be performed in the future for this technology to be really implemented in everyday practice. Also, training sessions in clinical settings could be of use, as many orthodontists are unaware of the benefits of this procedure. The results provided by the 19 studies included in this study show that the use of laser-technology for orthodontic bracket-removal is superior in terms of clinical results as it reduces bond strength, it facilitates the removal of remanent bracket adhesive, it prevents damage to the enamel and temperature variations that occur throughout the procedure can be controlled with careful device settings adjustments.

Looking back at the stated purposes of our study, we can make the following statements:

- While laser technology has been tested for the purpose of orthodontic bracket removal, out of the 405 references found in relation with the topic only 19 studying the application of laser technology during the actual step of debonding were eligible for review and all of them were in-vitro studies. Only two of the studies used laser-technology to debond metallic brackets, both of them using Er:YAG lasers.
- All types of lasers used in the 19 studies have proven to be effective for bracket removal, however studies with Er:YAG technology have shown better results in preserving enamel integrity and controlling the intra-pulpal temperature variation within a safe range to preserve pulp vitality. Er:YAG lasers with energy levels of 4 W combined with water cooling spray using a scanning mode have been shown to be safe for bracket debonding, although maybe not entirely time-efficient as it requires 6 s per tooth.
- The use of lasers for orthodontic bracket removal has the advantage of decreasing the force necessary for debonding and most times decreasing the amount of remanent adhesive, thus protecting the enamel. Although the use of laser-technology increases the intra-pulpal temperature, the adequate parameters for each type of lasers should keep temperature variations within a safe limit.
- All laser technologies presented in the eligible studies have proven to be effective by reducing the bond strength between bracket and substrate, however, when softening of the adhesive occurs it is important that also the quantity of remanent adhesive is as little as possible in order to protect the integrity of the enamel.

## Figures and Tables

**Table 1:** Results of articles using CO_2_ laser technology.

Author	Year	Type of Study	Type of LASER Used	Type of Bracket Used	Results/Conclusion
Ma et al.	1997	Comparative Laser/No Laser	CO_2_	Ceramic	Significant statistical difference (*p* < 0.05) was found between the tensile strength needed for debonding between the control group and the experimental group. Authors stated that debonding of ceramic brackets using laser technology while also avoiding an increase of the intra-pulpal temperature beyond the threshold of pulpal damage is feasible [21].
Tehranchi et al.	2011	Comparative Laser/No Laser	super pulse CO_2_ laser	Ceramic	The debonding site in the control group was closer to the enamel adhesive interface and, the rate of enamel damage in this group was greater. The use of super pulse CO_2_ laser diminishes significantly the debonding force and increases the amount of remanent adhesive on the tooth surface [26].
Ahrari et al.	2012	Comparative Laser/No Laser	CO_2_	Ceramic	Laser-technology used for debonding of ceramic brackets can: decrease the risk of damage to the enamel reduce the risk of bracket fracture modify the sores of the Adhesive Remanent Index (ARI) towards a more favorable outcome prevent causing thermal damage to the pulp [14].
Macri et al.	2015	Comparative Laser settings	CO_2_ laser irradiation with different regimens	Ceramic	CO_2_ laser irradiation 1 0 W, 0.01 s, 3 s regimen was one in which the strength of debonding is 7.33 MPa. CO_2_ laser can be used for debracketing as it decreased the bond strength of the adhesive without increasing the temperature excessively [27].
Saito et al.	2015	Comparative between different time exposures to laser in association with bonding materials containing various microcapsule contents (0, 30, and 40 wt%)	CO_2_	Ceramic	CO_2_ laser technology in combination with a orthodontic adhesives containing thermal expansion microcapsules can be used effectively for debonding ceramic brackets. This combination is safe to use as it produces less enamel damage and no dental pain [28].

**Table 2:** Articles using Er:YAG technology.

Author	Year	Type of Study	Type of LASER Used	Type of Bracket Used	Results/Conclusion
Mundethu et al.	2014	Experimental	Er:YAG laser emitting a wavelength of 2.94 μm	Ceramic	The debracketing was in most cases due to thermomechanical ablation in the superficial part of the adhesive layer. The bracket flipped from the tooth without any additional force. Light microscopy and SEM emphasized the lack of damages to the enamel surface [29].
Dostalova et al.	2016	Comparative Laser/No Laser	Er:YAG laser 280 mJ, 250 s long, repetition rate 6 Hz, spot focus 1 mm, and 140 s.	Ceramic and metallic	Bracket removal was proven to require less work/force after the Er:YAG laser irradiation, while the temperature rise during the procedure was limited (from 2.0 °C to 3.2 °C). The findings are similar in case of use of metallic brackets. Samples where laser technology was used presented no damage to the enamel during SEM investigations [30]
Hamadah et al.	2016	Comparative in regard with pulse duration for the laser	Er:YAG laser for 6 s by laser scanning method	Ceramic	The debonding of ceramic brackets using the ER:YAG Laser technology with scanning lasing method is effective and feasible without additional risks if the pulse durations are of 100 and 300 μs [31].
Grzech-Lesniak et al.	2018	Comparative between scanning and circular motion technique	Er:YAG laser wavelength of 2940 nm at a power of 3.4 W, energy 170 mJ, frequency 20 Hz, pulse duration 300 μs, tip diameter 0.8 mm, air/fluid cooling 3 mL/s, and time of irradiation: 6 s.	Ceramic and metallic	By comparing the scanning and circular motion methods using the Er:YAG laser on ceramic brackets, it was observed that the former causes a significantly (*p* = 0.0001) lower temperature increase (mean: 0.83 °C in comparison with mean: 1.78 °C). Also on the metal brackets (mean: 1.29 °C; *p* = 0.015) the same phenomena was described. Er:YAG lasing during debonding procedures bring a slight increase in the pulp temperature but provide a reduced the risk of enamel damage compared with mechanical bracket debonding techniques [22].
Nalbantgil et al.	2018	Comparative between different energy levels	ER:YAG laser with 2, 4, or 6 Watt energy levels	Ceramic	Mean Temperature increases and respectively Mean Shear Bond Strengths were as follows: Control: 0, respectively 21.35 ± 3.43 ER:YAG-2Watt: 0.67 °C ± 0.12 °C, 8.79 ± 2.47, ER:YAG-4Watt: 1.25 °C ± 0.16 °C, 3.28 ± 0.73 ER:YAG-6Watt: 2.36 °C ± 0.23, 2.46 ± 0.54 4 watts energy level with water cooling spray for 6 sec in scanning mode with was concluded to be the most efficient and safe when using the Er:YAG laser during debonding [23].

**Table 3:** Studies using Nd:YAG, Diode and fiber-laser Technologies.

Author	Year	Type of Study	Type of LASER Used	Type of Bracket Used	Results/Conclusion
Hayakawa et al.	2005	Comparative single-/poly-crystalline bracket, different adhesives	high-peak power Nd:YAG laser	Ceramic	The use of a high-peak power Nd:YAG laser at 2.0 J or more is considered to be effective for debonding ceramic brackets [32].
Han et al.	2008	Comparative of Shear Bond Strength between Metallic brackets, Ceramic brackets, Ceramic brackets debonded with Laser	Nd:YAG laser at 1060 nm, pulse width of 0.2 ms, and 3 W for 3 s	Ceramic and metallic	The use of Nd:YAG laser can be effective in reducing the necessary debonding force, can determine the appearance of less remnant adhesive, and decreases the risk of enamel damage [33].
Dostalova et al.	2011	Comparative between two power settings for the laser	diode-pumped (Tm:YAP) microchip laser at a wavelength of 1998 nm with two power settings (1–2 W)	Ceramic	Use of a Tm:YAP laser (wavelength 1998 nm, power 1 W, irradiance 14 W/cm^2^, interacting time 60 s) with moderate cooling, could be an efficient tool for debracketing [34].
Sarp et al.	2011	Comparative between continuous and modulated mode	A new fiber laser (1070-nm ytterbium fiber laser)	Ceramic	Significant statistical differences were found between the experimental and the control groups in regard with the necessary debonding force, time, and work done by a universal testing machine. For the experimental groups, the three measured parameters were reduced. A proper setting of the Laser parameters can bring 50% of reduction in required load for debonding and three fold decrease in debonding time were observed. In the continuous mode, with energy levels inferior to 3.5 W, the temperature changes in the pulpal chamber were below the accepted threshold value (5.5 °C), also, the work done by the testing machine in order to cause the debonding was decreased up to 5 times. While comparing the continuous and modulated mode of application, it was observed that with the modulated mode debracketing appeared faster and easier, with less temperature change [35].
Stein et al.	2017	Comparative Laser/No Laser	445-nm diode laser	Ceramic	Lasing with the 445nm diode laser prior to debonding the ceramic brackets from the tooth surface favors an adhesive failure with less remaining adhesive on the dental structures. This is important for the clinicians as it reduces chair time during debonding and also reduces enamel damage [36].

**Table 4:** Comparative Studies between different types of laser technologies.

Author	Year	Type of Study	Type of LASER Used	Type of Bracket Used	Results/Conclusion
Strobl et al.	1992	Comparative between lasers and mono-/polycrystalline alumina brackets	CO_2_ laser wavelength (10.6 I-tm), YAG laser frequency of 1.06 g.m	Ceramic	The laser-debonding techniques showed: a need for less force needed for debonding a low risk in damaging the enamel a reduced incidence of failure when compared with the purely mechanical debonding techniques. The clinical significance of the method being that it is less traumatic (less painful) for the patient and is safer (less risk of enamel damage) [37].
Tocchio et al.	1993	Comparative between different lasers and monocrystalline/polycrystalinebrackets	XeCI excimer laser, operating at 308 nm, 8 W, KrF at 248 wavelength, Nd:YAG at a 1060 wavelength	Ceramic	For polycrystalline brackets. Debonding occurs by bracket slide off indicating a process of thermal softening within the resin. High temperatures are suspected to arise during debonding because of long debonding times, carbon deposits and porosity on the debonding surface as well as high temperatures on the brackets For monocrystalline (sapphire) brackets. During debonding, little heating of the bracket occurs, as the light is transmitted as a coherent, focused beam through the brackets to the bonding interface The use of 248 and 308 nm radiation caused a phenomenon of bracket blow off, meaning that it is the ablation that causes debonding. With the use of 248 nm radiation it is impossible to know if the debonding was made by thermal or photo- ablation With the use of 308 nm radiation with more than 17 W/cm densities, the debonding time is 0.5 s at the most. The use of 9 W/cm z or lower densities makes thermal ablation responsible for debonding [38].
Jelinkova et al.	2009	Comparative between three lasers from different parts of the spectrum and tryout for different wevelengths and radiation power for debonding	Diode-pumped continuously running Tm:YAP, Nd:YAG lasers, GaAs laser diode generating radiation with the wavelengths 1.997 μm, 1.444 μm, and 0.808 μm, respectively	Ceramic	the 1.997 μm Tm:YAP and 1.444 μm Nd:YAG with the power 1 W acting 60 s are giving the reasonable dose for brackets tear off [39].
Hoteit et al.	2020	Comparative between laser setings	Er,Cr:YSGG of a 2780 nm wavelength, Er:YAG laser wavelength of 2940 nm	Ceramic	Improper adjustment of laser parameters may damage the enamel surface while debonding ceramic brackets even more than, conventional manual mechanical debonding. Using Er,Cr:YSGG (4 W/20 Hz) to debond orthodontic ceramic brackets enables the protection of enamel. While the use of Er,Cr:YSGG or Er:YAG will increase the microhardness of the enamel surface, its toughness to fracture will decrease, as they are indirectly proportional [40].

## Data Availability

All cited data is available within the text of the articles studied for this review.
